# Transcatheter hepatic arterial chemoembolization combined with Kangai injection for hepatitis B virus-related hepatocellular carcinoma: A protocol for a PRISMA-compliant meta-analysis

**DOI:** 10.1097/MD.0000000000022565

**Published:** 2020-10-09

**Authors:** Wanpeng Wang, Shurong Wang, Jia Liu, Yan Liu, Ying Mu, Jing Wang

**Affiliations:** a Department of infectious diseases, Weifang People's Hospital; b Quality Control Office, People's Hospital of Weifang Binhai Economic and Technological Development Zone; c Department of Gynaecology; d Department of Gastroenterology, Weifang People's Hospital, Weifang; e Department of Gastroenterology, Liaocheng People's Hospital, Liaocheng, Shandong Province, PR China.

**Keywords:** clinical symptoms, hepatitis B virus-related hepatocellular carcinoma, immune function, Kangai injection, quality of life

## Abstract

**Background:**

Kangai injection, a well-known insect-derived traditional Chinese medicine preparation, has been widely applied as a promising adjunctive drug for hepatitis B virus (HBV)-related hepatocellular carcinoma (HCC). However, its exact clinical efficacy and safety is still not well investigated. In this study, we aimed to summarize the efficacy and safety of Kangai injection for patients with HBV-related HCC through the meta-analysis.

**Methods:**

All available randomized controlled trials (RCTs) and high-quality prospective cohort studies that investigated the efficacy and safety of Kangai injection for patients with HBV-related HCC were searched from ten electronic databases including Google Scholar, PubMed, Excerpt Medica Database (Embase), Cochrane Library, Medline, Web of Science (WOS), China National Knowledge Infrastructure (CNKI), China Scientific Journal Database (CSJ) Chinese, Biomedical Literature Database (CBM) and Wanfang Database. Papers in Chinese or English published from January 2000 to September 2020 will be included without any restrictions.

Study selection and data extraction will be performed independently by 2 researchers. The clinical outcomes including overall response rate (ORR), disease control rate (DCR), quality of life (QoL), clinical symptoms, virological indicators, immune function and adverse events, were systematically evaluated. Review Manager 5.3 and Stata 14.0 were used for data analysis, and the quality of the literatures was also evaluated.

**Results:**

The results of this study will be published in a peer-reviewed journal, and provide a helpful evidence for clinicians to formulate the best postoperative adjuvant treatment strategy for HBV-related HCC patients.

**Conclusion:**

Our study will draw an objective conclusion of the efficacy of Kangai injection on curative effect (ORR and DCR), clinical symptoms, virological indicators, QoL, and immune function in patients with HBV-related HCC.

**INPLASY registration number:**

INPLASY202090014.

## 1 Introduction

Hepatocellular carcinoma (HCC) is the seventh most commonly diagnosed malignancy and the second leading cause of cancer-related death in 2018, with an incidence of 841,000 new cases and

782,000 deaths per year.^[1,2]^ Hepatitis B virus (HBV) infection is one of the main factors that causes HCC, and about 50% of HCC is attributed to HBV.^[3–6]^ According to the World Health Organization (WHO), HBV claimed 887,000 lives in 2015 and resulted in 257 million chronic carriers in 2017.^[5,7–9]^ The occurrence of chronic hepatitis B varies by geographic area, and the incidence rate is high in Africa and Asia.^[7,10,11]^ More than half HCC patients already have advanced or metastatic lesions when diagnosed, due to the lack of noticeable clinical symptoms.^[12]^ For these patients with advanced HCC, treatment options are limited to palliative treatment, such as antiviral therapy or transcatheter hepatic arterial chemoembolization (TACE).^[13–18]^ Unfortunately, the chemoradiotherapy regimens commonly used to treat HBV-related HCC often cause serious adverse effects, which severely affect the immune function and quality of life (QoL) of HBV-related HCC patients.^[12]^ Therefore, exploring new alternative regimens with better tolerance and lower toxicity for HBV-related HCC patients are urgently required.

Traditional Chinese medicine has been considered as a promising option to treat HBV-related HCC due to its unique biological characteristics, such as anti-tumor angiogenesis, induction of tumor cell apoptosis, immune regulation, and analgesia.^[19–24]^ Kangai injection, a famous insect-derived traditional Chinese medicine, was extracted from 3 Chinese herbs including *ginseng, astragalus membranaceus*, and *oxymatrine*.^[25,26]^ It contains many active ingredients such as *astragalus polysaccharides* and *astragalosides* (the major effective antitumor constituents of *astragalus*), *ginsenosides* and *ginseng polysaccharide* (the major effective antitumor constituents of *ginseng*) and *oxymatrine*.^[25,27]^ Kangai injection can exert the anti-tumor efficiency through a variety of mechanisms, including through inducing tumor cell apoptosis, inhibiting tumor cell proliferation, invasion and metastasis, and improving the bodys immune function.^[27–30]^ in addition, it can also can effectively increase the sensitivity of tumor cells to chemotherapeutic agents, and reduce the related adverse events caused by radiochemotherapy.^[27–30]^

**Figure 1:**
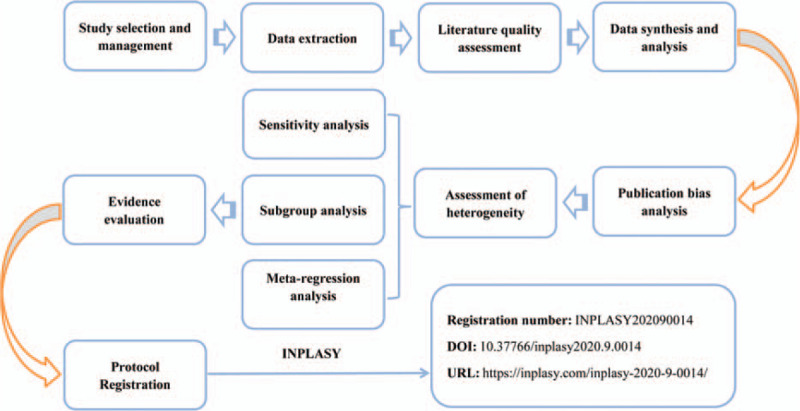
Work flow of the present study.

Currently, Kangai injection has attained great popularity in the alternative and complementary treatment of HBV-related HCC.^[29,31,32]^ Several studies have indicated that the combination of Kangai injection and classic radiochemotherapy not only exerts an enhanced therapeutic effect against HBV-related HCC, but also improve the quality of life (QoL) and immune function of patients.^[29,31,32]^ Despite the intensive clinical studies, its clinical efficacy remains controversial. In this study, we are prepared to summarize the efficacy of Kangai injection combined with TACE on curative effect, clinical symptoms, virological indicators, QoL and immune function in patients with HBV-related HCC through the meta-analysis, in order to provide a helpful evidence for clinicians to formulate the best postoperative adjuvant treatment strategy for HBV-related HCC patients (Fig. 1, Work flow of the present study).

**Review question:** Is Kangai injection effective on curative effect, clinical symptoms, virological indicators, QoL and immune function in patients with HBV-related HCC?

**Objective:** A systematic review and meta-analysis will be performed to systematically evaluate the efficacy and safety of Kangai injection for the treatment of patients with HBV-related HCC.

## 2 Methods

Our systematic review and meta-analysis protocol has been registered on the International Platform of Registered Systematic Review and Meta-Analysis Protocols (INPLASY). The registration number was INPLASY202090014 (DOI: 10.37766/inplasy2020.9.0014, https://inplasy.com/inplasy-2020-9-0014/). The protocol of this meta-analysis will be reported according to Preferred Reporting Items for Systematic Review and Meta-Analysis Protocols (PRISMA-P) guidelines.^[33]^

**Ethics:** This meta-analysis is a secondary research which based on some previously published data. Therefore, the ethical approval or informed consent was not required.

### 2.1 Search strategy

To perform a comprehensive and focused search, experienced systematic review researchers will be invited to develop a search strategy. The plan searched terms are as follows: “hepatitis B virus” or “HBV” or “liver cancer” or “hepatocellular carcinoma” or “hepatitis B virus-related liver cancer” or “hepatitis B virus-related hepatocellular carcinoma” or “HBV-related liver cancer” or “HBV-related hepatocellular carcinoma” or “LC” or “HCC” and “transcatheter hepatic arterial chemoembolization” or “transcatheter arterial chemoembolization” or “TACE” et al. An example of search strategy for PubMed database shown in Table 1 will be modified and used for the other databases.

**Table 1:**
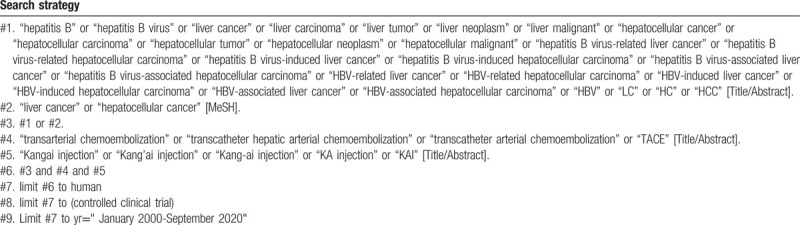
Searching strategy in PubMed.

### 2.2 Eligibility criteria

#### 2.2.1 Types of studies

All available randomized controlled trials (RCTs) or quasi-RCTs, and high-quality prospective cohort studies that investigated the efficacy and safety of Kangai injection for the treatment of patients diagnosed with HBV-related HCC will be included in this meta-analysis.

#### 2.2.2 Types of participants

Patients must be cytologically or pathologically confirmed as having HBV-related HCC according to the National Comprehensive Cancer Network (NCCN) Clinical Guidelines for HCC. Patients with other malignancies or non-HBV-related HCC are not included. No restrictions regarding age, gender, racial, region, education, and economic status in this analysis protocol.

#### 2.2.3 Types of interventions

HBV-related HCC patients in the experimental group must be treated with TACE combined with Kangai injection.

#### 2.2.4 Comparator

HBV-related HCC patient in the control group was treated with TACE only.

#### 2.2.5 Exclusion criteria

Papers without sufficient available data, non-comparative clinical trials, non-peer reviewed studies, literature reviews, meta-analysis, meeting abstracts, case reports, letter to the editor, and other unrelated researches will be excluded from analysis.

### 2.3 Information sources

Electronic databases including Google Scholar, PubMed, Excerpt Medica Database (Embase), Cochrane Library, Medline, Web of Science (WOS), China National Knowledge Infrastructure (CNKI), China Scientific Journal Database (CSJ) Chinese, Biomedical Literature Database (CBM) and Wanfang Database will be systematically searched for eligible clinical trials from January 2000 to September 2020. Language is limited with English and Chinese.

### 2.4 Types of outcome measures

#### 2.4.1 Primary outcomes

- Overall response rate (ORR, complete response + partial response) and disease control rate (DCR, complete response + partial response + stable disease);
- QoL as evaluated by Karnofsky score;
- Clinical symptoms, such as abdominal pain and distension, fatigue, and loss of appetite;
- Virological indicators: Quantitative detection of HBV-Deoxyribonucleic acid (HBV-DNA) and hepatitis B e antigen (HBeAg).

#### 2.4.2 Secondary outcomes

Secondary outcomes will include:

- Immune function indicators: CD3^+^, CD4^+^, CD8^+^, natural killer (NK) cells percentage, and CD4+/CD8+ cell ratios, and serum cytokines level [Interleukin-2 (IL-2), Interleukin-4 (IL-4), Interferon-γ (IFN-γ) and tumor necrosis factor-α (TNF-α)];
- Adverse events: toxicity was graded from 0 to IV in severity on the basis of the WHO recommendations.

### 2.5 Data collection and analysis

#### 2.5.1 Study selection and management

**Figure 2:**
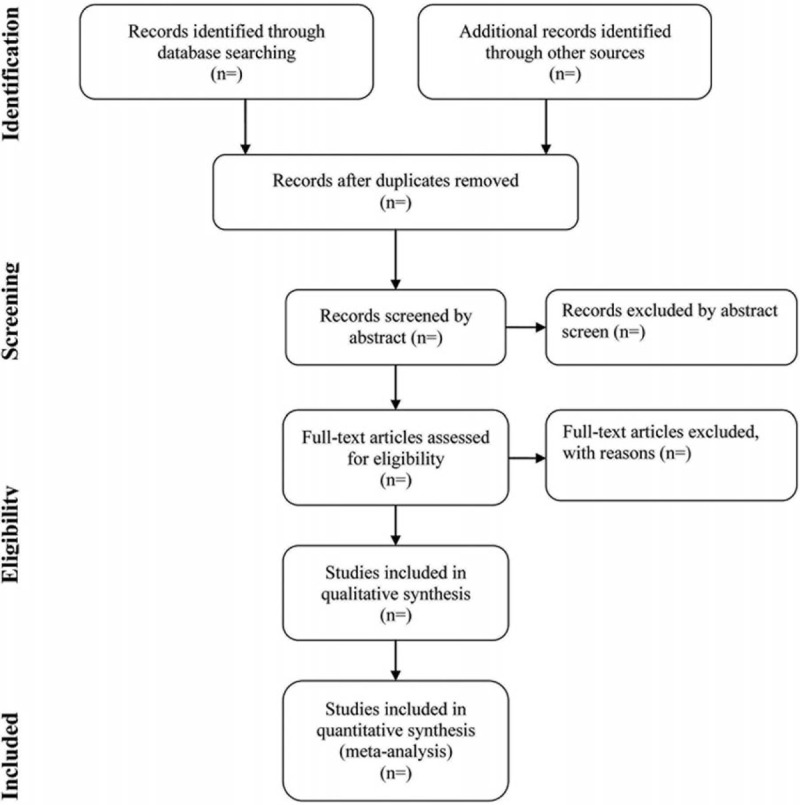
Study selection process for the meta-analysis.

Endnote X9 software will be used for literature managing and records searching. Two authors (Wanpeng Wang and Shurong Wang) will be reviewed independently to identify potential trials by assessing the titles and abstracts and identify whether the trials meet the inclusion criteria. The full text will be further reviewed to exclude irrelevant studies or determine potential eligible studies. If there is any disagreement, it will be resolved by consulting with the third investigator (Jia Liu). A PRISMA-compliant flow chart (Fig. 2) will be used to describe the selection process of eligible literatures.

#### 2.5.2 Data extraction and management

Two investigators (Wanpeng Wang and Shurong Wang) will be responsible for the data extraction independently according to the Cochrane Handbook for Systematic Reviews of Intervention.

The following data will be extracted from eligible literatures:

**Study characteristics and methodology:** country of study, the first authors name, year of publication, randomization, and follow-up duration, et al.

**Participant characteristics:** tumor stage, sample size, age, gender, ethnicity, pathology diagnosis, pathologic tumor size, inclusion, and exclusion criteria, et al.

**Interventions:** therapeutic means, dosage of Kangai injection, administration route and cycles, and duration of treatment, et al.

**Outcome and other data:** ORR, DCR, QoL, clinical symptoms, virological indicators, immune indexes, and adverse effects, et al.

**Dealing with missing data:** we will attempt to contact the authors via email to request the missing or incomplete data. If those relevant data are not acquired, we will use the available data for data synthesis.

### 2.6 Literature quality assessment

Two review investigators (Wanpeng Wang and Shurong Wang) will be assessed risk of bias of the included RCTs by independently based on the Cochrane bias risk tool. There are 7 items in total, including: random sequence generation, allocation concealment, blinding of participants and personnel, blinding of outcome assessment, incomplete outcome data, selective reporting, and other bias.^[34,35]^ Evidence quality will be classified as low risk, high risk, or unclear risk of bias. EPOC guidelines will be used to assess the risks of non-RCTs.^[36]^ Any disagreements will be resolved via discussion with a third researcher (Jia Liu).

### 2.7 Assessment of heterogeneity

Heterogeneity between studies will be assessed using the Cochran's Q and Higgins *I*^*2*^ statistic. *P* < .1 for the Chi^2^ statistic or an *I*^*2*^ > 50% will be considered as showing considerable heterogeneity.^[37]^ A fixed effect model will be used to calculate the outcomes when statistical heterogeneity is absent; otherwise, the random effects model will be used for analysis.^[38]^

### 2.8 Data synthesis

We will utilize Review Manager 5.3 (Nordic Cochran Centre, Copenhagen, Denmark) and Stata 14.0 (Stata Corp., College Station, TX, USA) statistical software to pool the data and carry out the data analysis. For continuous data, the extracted data will be presented as standardized mean difference (SMD) with their confidence intervals (CIs). Dichotomous data will be recorded as risk ratio (RR) with 95% CIs. A two-tailed *P* < .05 was considered statistically significant.

### 2.9 Subgroup and meta-regression analysis

If the included studies are sufficient (at least 10 trials), subgroup and meta-regression analysis will be conducted to explore the source of heterogeneity with respect to tumor stage, region, course of treatment, and therapeutic regimens, et al.

### 2.10 Sensitivity analysis

Sensitivity analysis will be carried out to assess the reliability and robustness of the pooled results via eliminating trials with low quality. A summary table will report the results of the sensitivity analyses.

### 2.11 Other relevant information

#### 2.11.1 Publication bias analysis

Funnel plot, Beggs, and Egger regression test will be performed to analyze the existence of publication bias if 10 or more studies are included in the meta-analysis.^[12,39–41]^ If reporting bias is suspected, we will consult the study author to get more information. If publication bias existed, a trim-and-fill method should be applied to coordinate the estimates from unpublished studies, and the adjusted results were compared with the original pooled RR.^[12,42,43]^

#### 2.11.2 Evidence evaluation

We will use the Grading of Recommendations, Assessment, Development, and Evaluation (GRADE) to assess the quality of evidence and the strength of the main result recommendations. The quality of all evidence will be evaluated as 4 levels: high, moderate, low, and very low.^[34]^

### 2.12 Dissemination plans

We will disseminate the results of this systematic review by publishing the manuscript in a peer-reviewed journal.

## 3 Discussion

Clinical trials have indicated that traditional Chinese medicine could significantly improve the efficiency of conventional treatments and reduces related toxicity, and plays an irreplaceable role in clinical practice.^[19–24,44,45]^ Many scholars pointed out that the combination of Chinese and Western medicine for HBV-related HCC may be the potential trend of clinical treatment in the future.^[29,31,32]^ Kangai injection is a famous insect-derived traditional Chinese medicine preparation that manufactured by Changbaishan Pharmaceutical Co., Ltd. It have been approved by Chinese State Food and Drug Administration (SFDA), and granted the Manufacturing Approve Number accordingly (Z20026868). Currently, Kangai injection has been applied alone or combined with conventional methods to treat various malignant tumors in China.^[27–30]^

### 3.1 Strengths and limitations

Even though there was statistical analysis of published clinical trials, the exact effects of Kangai injection for patients with HBV-related HCC were still not systematically investigated. This systematic review will conduct a systematic, comprehensive and objective evaluation of Kangai injection-based adjuvant therapy. The findings of this analysis will provide a helpful evidence for clinicians to formulate the best postoperative adjuvant treatment strategy for patients with advanced HBV-related HCC, and also provide scientific clues for researchers in this field. There may be a language bias with the limitation of English and Chinese studies. In addition, different tumor stage and age of patients, doses of Kangai injection among included trials may lead to significant clinical heterogeneity.

## Author contributions

**Conceptualization:** Wanpeng Wang, Jing Wang.

**Data curation:** Wanpeng Wang, Shurong Wang, Jia Liu, Yan Liu.

**Formal analysis:** Wanpeng Wang, Shurong Wang, Jia Liu, Yan Liu.

**Funding acquisition:** Ying Mu.

**Investigation:** Wanpeng Wang, Shurong Wang, Jia Liu, Yan Liu, Ying Mu.

**Methodology:** Wanpeng Wang, Shurong Wang, Jia Liu, Yan Liu, Ying Mu.

**Project administration:** Wanpeng Wang, Jing Wang.

**Resources:** Wanpeng Wang, Jing Wang.

**Software:** Wanpeng Wang, Jing Wang.

**Supervision:** Wanpeng Wang, Jing Wang.

**Validation:** Wanpeng Wang, Jing Wang.

**Visualization:** Wanpeng Wang, Shurong Wang, Jia Liu, Yan Liu.

**Writing – original draft:** Wanpeng Wang, Shurong Wang, Jia Liu, Yan Liu.

**Writing – review & editing:** Wanpeng Wang, Ying Mu, Jing Wang.

## Abbreviations

Abbreviations: CBM = Chinese Biomedical Literature Database, CIs = confidence intervals, CNKI = China National Knowledge Infrastructure, CSJ = China Scientific Journal Database, DCR = disease control rate, DNA = deoxyribonucleic acid, Embase = Excerpt Medica Database, GRADE = Grading of Recommendations, Assessment, Development, and Evaluation, HBeAg = hepatitis B e antigen, HBV = hepatitis B virus, HCC = hepatocellular carcinoma, IFN-γ = interferon-γ, IL-2 = interleukin-2, IL-4 = interleukin-4, INPLASY = International Platform of Registered Systematic Review and Meta-Analysis Protocols, NCCN = National Comprehensive Cancer Network, NK = natural killer, ORR = overall response rate, PRISMA-P = Preferred Reporting Items for Systematic Reviews and Meta-Analyses Protocols, QoL = quality of life, RCTs = randomized controlled trials, RR = risk ratio, SFDA = State Food and Drug Administration, SMD = standardized mean difference, TACE = Transcatheter hepatic arterial chemoembolization, TNF-α = tumor necrosis factor-α, WHO = World Health Organization, WOS = Web of Science.
